# Crosstalk between the HIF-1 and Toll-like receptor/nuclear factor-κB pathways in the oral squamous cell carcinoma microenvironment

**DOI:** 10.18632/oncotarget.9329

**Published:** 2016-05-12

**Authors:** Shengwei Han, Wenguang Xu, Zhiyong Wang, Xiaofeng Qi, Yufeng Wang, Yanhong Ni, Hao Shen, Qingang Hu, Wei Han

**Affiliations:** ^1^ Department of Oral and Maxillofacial Surgery, Nanjing Stomatological Hospital, Medical School of Nanjing University, Nanjing, P.R. China; ^2^ Central Laboratory of Stomatology, Nanjing Stomatological Hospital, Medical School of Nanjing University, Nanjing, P.R. China

**Keywords:** oral squamous cell carcinoma, HIF-1, TLR, NF-κB, tumor microenvironment

## Abstract

Hypoxia is a prominent feature of the microenvironment of solid tumors and may contribute to tumor progression through the oxygen-sensitive transcriptional regulator hypoxia-inducible factor-1 (HIF-1). Chronic inflammation is another typical feature. Inflammatory mediators, including Toll-like receptors (TLRs) and nuclear factor-κB (NF-κB), play an important role in cancer development. Recent studies have revealed extensive cross-talk between hypoxia and inflammation signaling, though the mechanisms remain unclear. Our results confirm that *TLR3* and *TLR4* are highly expressed in oral squamous cell carcinoma (OSCC). Activation of TLR3 and TLR4 stimulated the expression of *HIF-1* through NF-κB. In addition, HIF-1 increased the expression of *TLR3* and *TLR4* through direct promoter binding. Thus, the TLR/NF-κB pathway forms a positive feedback loop with HIF-1. These results indicate a novel cross-talk between the TLR/NF-κB and HIF-1 signaling, which may contribute to OSCC initiation and progression. With the elucidation of this novel mechanism, it might serve as a basis for future microenvironment targeted cancer therapy.

## INTRODUCTION

Oral squamous cell carcinoma (OSCC) is one of the most common cancers of the head and neck region [1]. Despite advances in surgery, radiotherapy, and chemotherapy, the five-year survival rate is still 50–60% [2, 3]. Many strategies for the development of new OSCC therapeutics have considered the role of the tumor microenvironment in cancer development and progression [4]. In solid tumors such as OSCC, hypoxia and chronic inflammation are two of the most prominent features of tumor progression [5].

Hypoxia is generated by an insufficient blood supply during tumor growth [6]. The oxygen pressure within solid tumors is heterogeneous and can range from approximately 5% O_2_ in well-vascularized regions to 1% O_2_ (hypoxic conditions) near necrotic regions [7]. We previously demonstrated that hypoxia was associated with tumor progression and clinical prognosis in OSCC [8]. Hypoxia-inducible factor-1 (HIF-1), a key mediator of the cellular response to hypoxia, is comprised of an oxygen-regulated HIF-1α subunit and a constitutively expressed HIF-1β subunit [9]. The HIF-1 dimer regulates the expression of more than 100 downstream genes that protect cells under hypoxic conditions [10].

Inflammation is another aspect of the tumor microenvironment that has a significant role in tumor progression. Under normal conditions, the inflammatory response can promote tissue repair processes such as wound healing. However, an abnormal inflammatory response can ultimately lead to tumorigenesis [11]. In solid tumors, cell growth can exceed the oxygen and nutrient supply resulting in hypoxia. This promotes the release of pro-inflammatory mediators that recruit additional inflammatory cells [12]. Nuclear factor-κB (NF-κB) is a key transcription factor involved in the inflammatory response and is thought to be a critical link between inflammation and cancer [13]. NF-κB is expressed in almost all cell types and is involved in cellular responses to stimuli such as stress, cytokines, apoptosis, and immune reactions [14]. NF-κB signaling is mediated by Toll-like receptors (TLRs), interleukin-1 receptor (IL-1R), and the tumor necrosis factor receptor (TNFR). Inflammation and NF-κB in particular play dual roles in cancer progression. NF-κB activation is part of the immune response and is particularly involved in acute inflammatory processes. NF-κB is constitutively activated in many cancers and can exert a variety of pro-tumorigenic effects [15]. Activation of NF-κB typically results in upregulation of anti-apoptotic genes and activation of cell survival mechanisms [16]. Cancer-related inflammation is a potential target for innovative therapeutic strategies. Therefore, NF-κB suppression could have therapeutic potential. Both *in vitro* and *in vivo* studies have shown that targeted NF-κB inhibition sensitized tumor cells to chemotherapy and radiation. However, targeting NF-κB in solid tumors did not achieve optimal treatment outcomes [17]. This may be explained by the tumor microenvironment. Despite inhibition of inflammatory signaling pathways, other pathways could still stimulate NF-κB expression. Recently, hypoxia in a solid tumor microenvironment was shown to activate NF-κB signaling [18]. However, the mechanisms by which hypoxia regulates NF-κB activity are unclear.

TLRs are a family of transmembrane receptors that recognize conserved microbial structures/patterns. It was initially though that TLRs were only expressed in immune cells and that they played a key role in the host defense against infection by recognizing a range of chemicals produced by bacteria, viruses, fungi, and protozoa [19]. However, recent data has indicated that various cancer cells also express TLRs. To date, 13 mammalian TLRs have been described, 11 of which are expressed in humans. *TLR3, TLR4*, and *TLR9* are found in breast, prostate, and colon cancer [20, 21, 22]. *TLR7* expression has also been reported in esophageal squamous cell carcinoma [23]. Finally, *TLR3* and *TLR4* expression was observed in head and neck squamous cell carcinoma (HNSCC) [24, 25]. TLRs are functionally active in various tumors and might induce cancer cell resistance to apoptosis [26].

In the present study, we demonstrated that TLR signaling induced *HIF-1* expression via NF-κB. Moreover, HIF-1, as part of a transcriptional response to hypoxia, directly activated the TLR/NF-κB signaling pathway in OSCC. Our results demonstrate that HIF-1 and TLR/NF-κB form a positive feedback loop in OSCC cells that connects hypoxia to inflammation, which contributes to OSCC initiation and progression.

## RESULTS

### TLRs are highly expressed in OSCC

**Figure 1:**
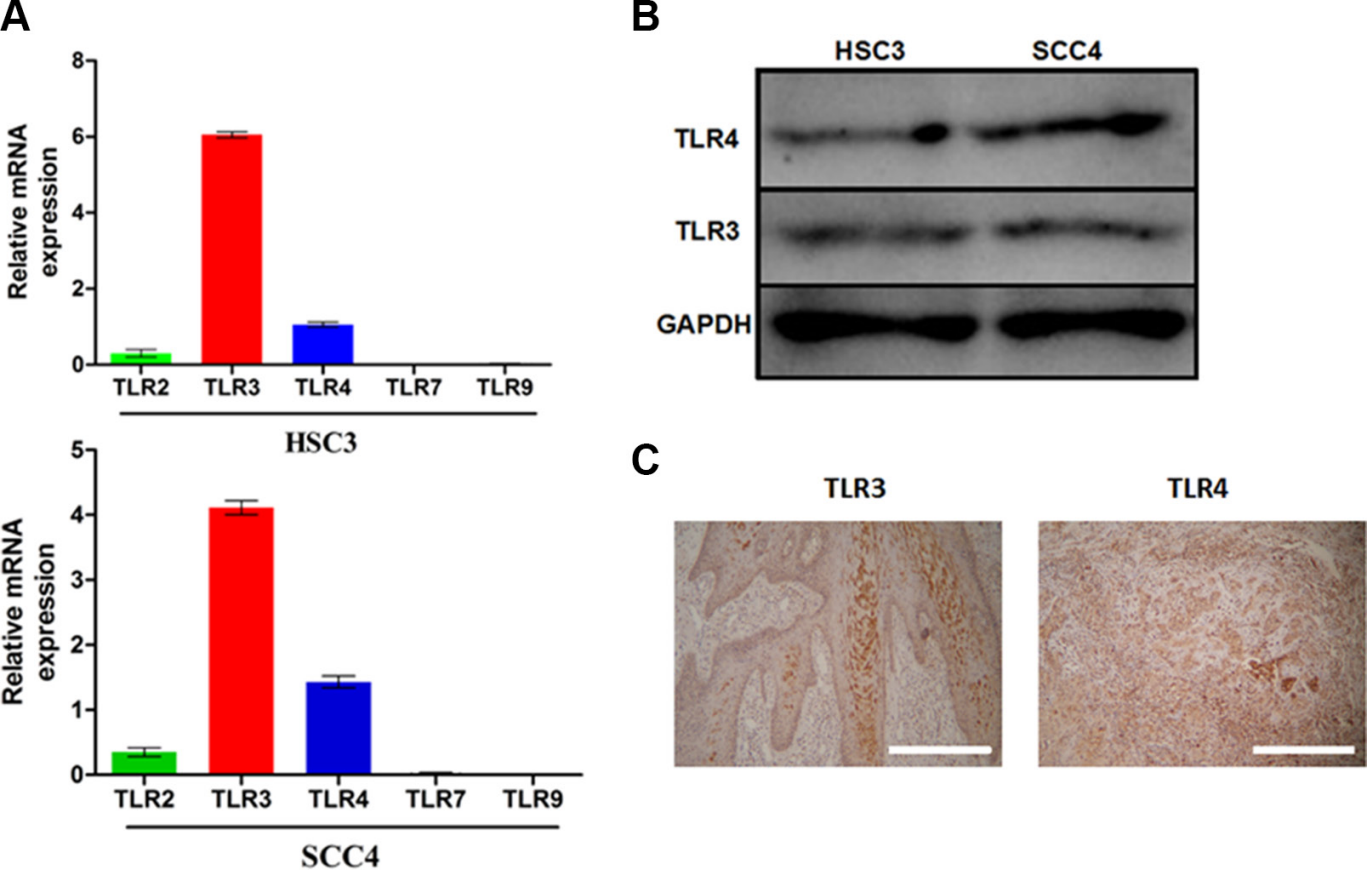
TLR3 and TLR4 are expressed in OSCC (**A**) Relative mRNA expression of *TLR2*, *TLR3*, *TLR4*, *TLR7*, and *TLR9* in the SCC4 and HSC3 OSCC cell lines. (**B**) Expression of TLR3 and TLR4 in HSC3 and SCC4 cells. (**C**) Expression of TLR3 and TLR4 in tumor tissue from OSCC patients (200×).

We investigated the expression of *TLR2*, *TLR3*, *TLR4*, *TLR7*, and *TLR9* in two OSCC cell lines (HSC3 and SCC4). Quantitative reverse transcription-PCR (qRT-PCR) revealed that these two cell lines expressed higher levels of *TLR3* and *TLR4* mRNA than *TLR2, TLR7*, and *TLR9* (Figure 1A). TLR3 and TLR4 expression in these cells was confirmed by western blotting (Figure 1B). High levels of TLR3 and TLR4 were also observed in OSCC patient samples as shown by immunohistochemistry (Figure 1C).

### TLRs induce *HIF-1* expression in OSCC cells

**Figure 2:**
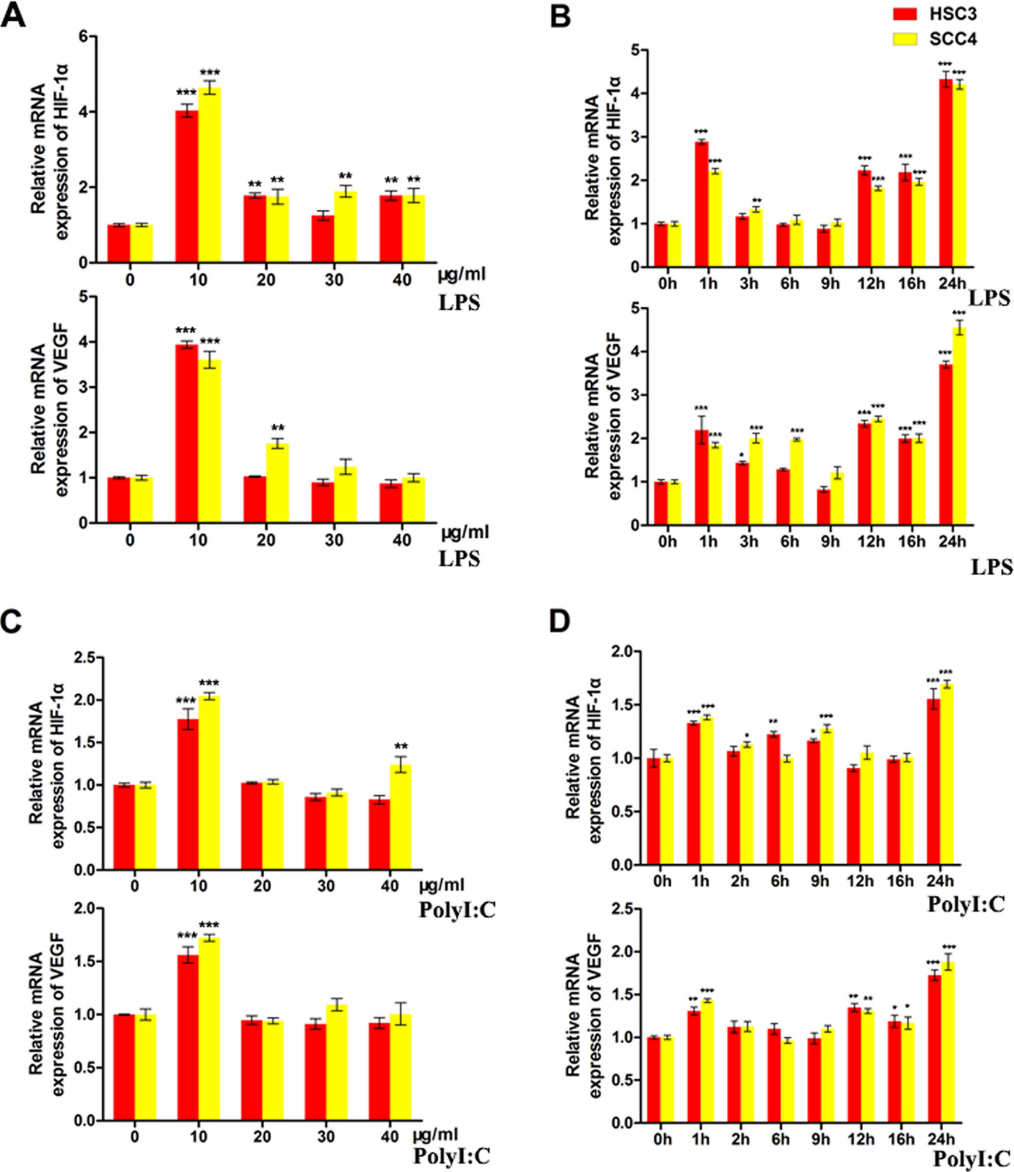
LPS and poly (I:C) induce *HIF-1α* and *VEGF* expression in HSC3 and SCC4 cells (**A**) Relative mRNA expression of *HIF1A* and its target gene *VEGF* in HSC3 and SCC4 cells treated with 0–40 μg/mL LPS for 24 h. (**B**) Relative mRNA expression of *HIF1A* and its target gene *VEGF* in HSC3 and SCC4 cells treated with 10 μg/mL LPS for 0–24 h. (**C**) Relative mRNA expression of *HIF1A* and its target gene *VEGF* in HSC3 and SCC4 cells treated with 0–40 μg/mL poly (I:C) for 24 h. (**D**) Relative mRNA expression of *HIF1A* and its target gene *VEGF* in HSC3 and SCC4 cells treated with 10 μg/mL poly (I:C) for 0–24 h. Error bars indicate SE (*p ≤ 0.05; **p ≤ 0.01; ***p ≤ 0.001).

We next examined whether TLR4 pathway activation could alter the expression of *HIF-1* in OSCC cells. Our results indicated that lipopolysaccharide (LPS), a TLR activator, induced the expression of *HIF-1α* and its target gene vascular endothelial growth factor (*VEGF*) in both HSC3 and SCC4 cells in a dose- and time-dependent manner. Maximum induction was observed after treatment with 10 μg/mL LPS for 24 h (Figure 2A–2B). Polyinosinic-polycytidylic acid [poly (I:C)] (10 μg/mL), a TLR3 ligand, induced expression of *HIF-1α* and *VEGF* in HSC3 and SCC4 cells in a time-dependent manner (Figure 2C–2D), suggesting that TLR pathway activation resulted in upregulation of *HIF-1α* and *VEGF* expression.

**Figure 3:**
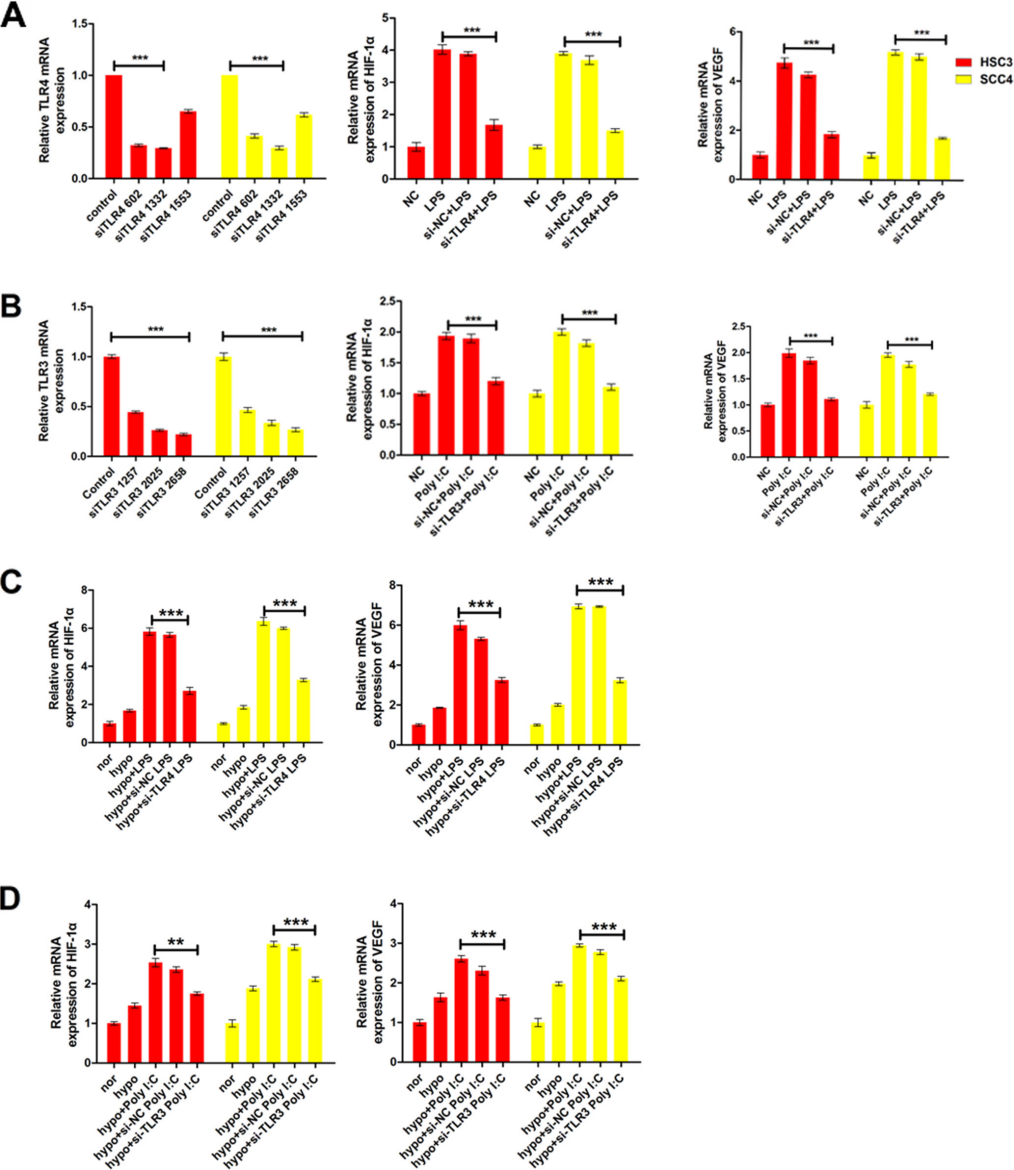
LPS and poly (I:C) induce HIF-1α and VEGF expression *via* TLR3 or TLR4 (**A**) Relative mRNA expression of *HIF1A* and its target gene *VEGF* in HSC3 and SCC4 cells transfected with 20 nM siTLR4 1332 prior to treatment with 10 μg/mL LPS. (**B**) Relative mRNA expression of *HIF1A* and its target gene *VEGF* in HSC3 and SCC4 cells transfected with 40 nM siTLR3 2658 prior to treatment with 10 μg/mL poly (I:C). (**C**) Relative mRNA expression of *HIF1A* and its target gene *VEGF* under hypoxic conditions (1% O_2_). Cells transfected with 20 nM siTLR4 1332 prior to treatment with 10 μg/mL LPS. (**D**) Relative mRNA expression of *HIF1A* and its target gene *VEGF* under hypoxic conditions (1% O_2_). Cells transfected with 40 nM siTLR3 2658 prior to treatment with 10 μg/mL poly(I:C) treatment. Error bars indicate SE (*p ≤ 0.05; **p ≤ 0.01; ***p ≤ 0.001). NC, negative control.

To further investigate whether TLRs regulated *HIF-1α* and *VEGF* expression, we knocked down *TLR3* and *TLR4* using three independent small interfering RNAs (siRNAs). Treatment of the cells with an anti-TLR4 siRNA (siTLR4 1332) decreased *TLR4* mRNA levels by > 70% in both HSC3 and SCC4 cell lines (Figure 3A). Concomitantly, this siRNA inhibited LPS-induced *HIF-1α* and *VEGF* expression (Figure 3A). Similarly, an siRNA that decreased *TLR3* expression by 70% (siTLR3 2658) also resulted in a significant reduction in poly (I:C)- induced *HIF-1α* and *VEGF* expression in HSC3 and SCC4 cells (Figure 3B). Our results also demonstrated that hypoxia enhanced LPS- and poly (I:C)-induced *HIF-1α* and *VEGF* expression, which was decreased by treatment with siRNAs targeting TLR3 and TLR4 (Figure 3C–3D). These results strongly suggested that TLR3 and TLR4 signaling was involved in regulation of *HIF-1α* and *VEGF* expression in OSCC cell lines.

### NF-κB is involved in TLR3- and TLR4-mediated regulation of *HIF-1*

**Figure 4:**
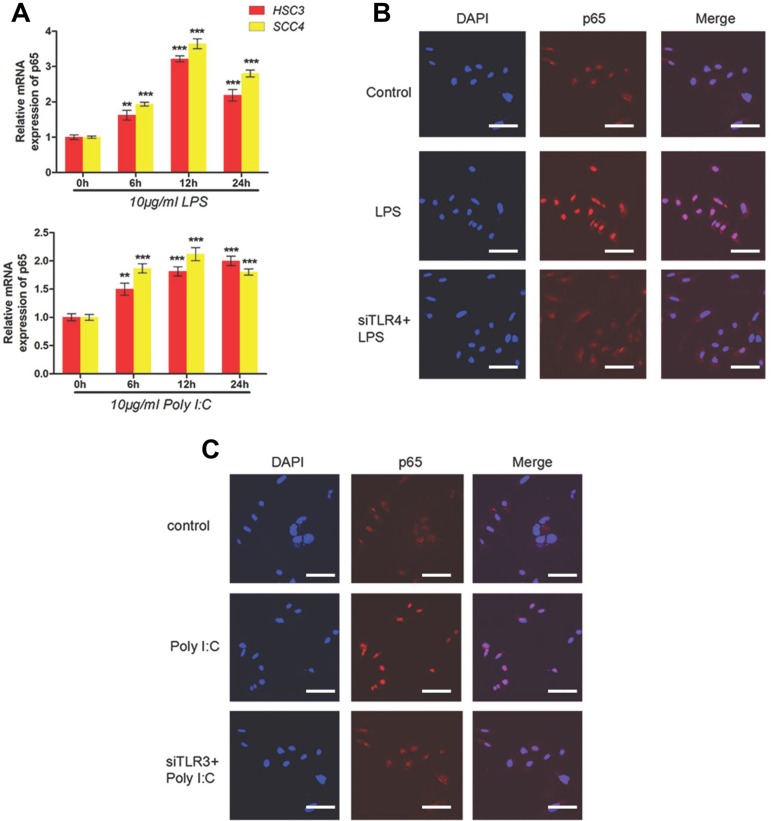
LPS and poly (I:C) induce HIF-1α and VEGF expression via the TLR-NF-κB pathway in OSCC (**A**) Relative mRNA expression of *p65* in HSC3 and SCC4 cells treated with 10 μg/mL LPS or poly (I:C) for 0–24 h. (**B**) Localization of p65 in cells treated with 10 μg/mL LPS for 2 h with or without transfection of siTLR4. Blue, nuclei; Red, p65. (**C**) Localization of p65 in cells treated with 10 μg/mL poly(I:C) for 2 h with or without transfection of siTLR3. Blue, nuclei; Red, p65. Error bars indicate SE (*p ≤ 0.05; **p ≤ 0.01; ***p ≤ 0.001).

Given that NF-κB plays a key role in the TLR pathway, we investigated the possible role of NF-κB in TLR-dependent upregulation of *HIF-1* using qRT-PCR. These results confirmed increased expression of the p65 subunit of NF-κB after treatment with LPS or poly (I:C) (Figure 4A). Using immunofluorescence, we demonstrated that treatment of HSC-3 cells with LPS and poly (I:C) led to the nuclear localization of p65 and activation of NF-κB signaling, which was not observed in cells transfected with TLR4 siRNA (Figure 4B–4C).

**Figure 5:**
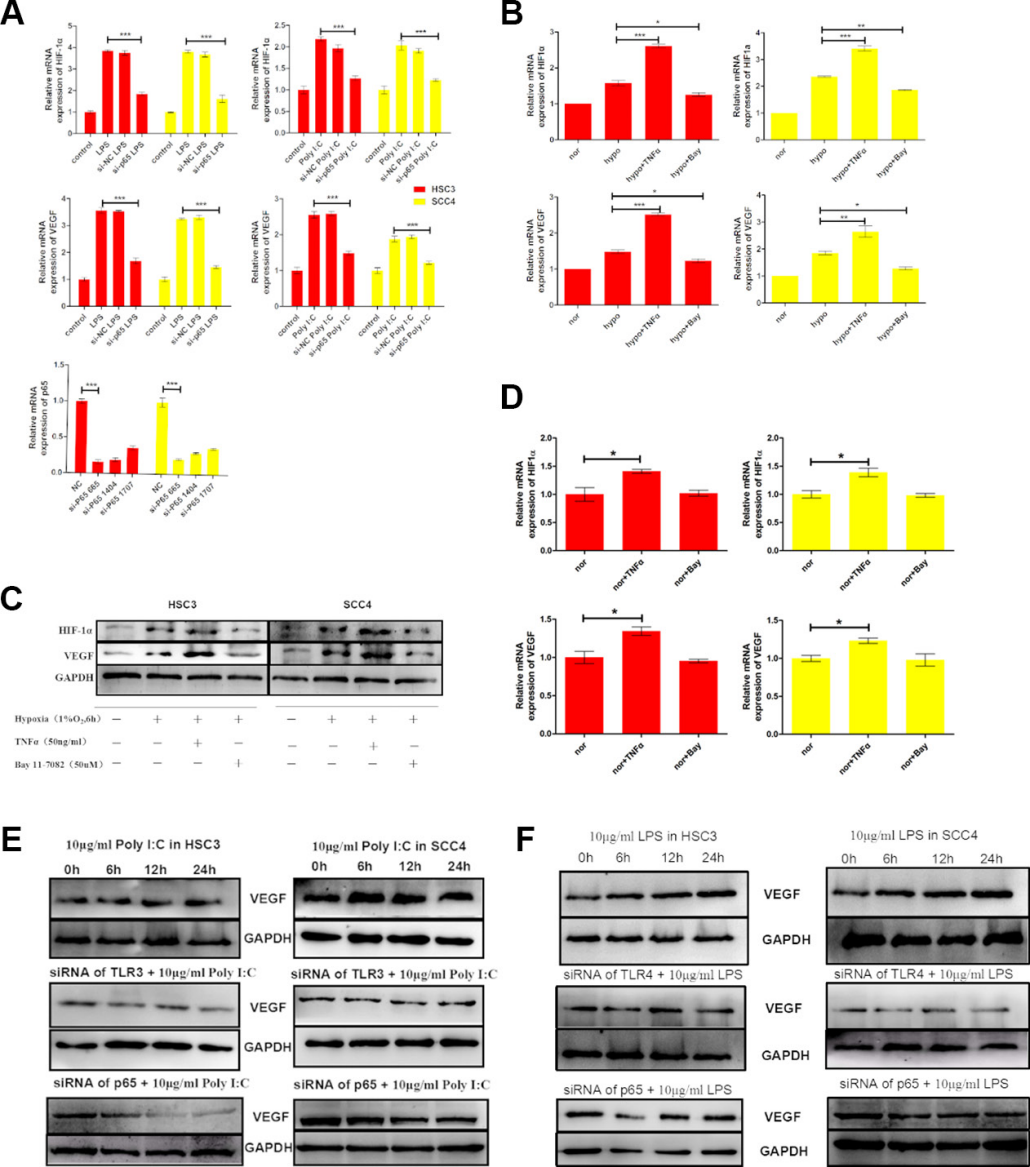
The TLR-NF-κB pathway regulates HIF-1α and VEGF expression in HSC3 and SCC4 cells (**A**) (Bottom) Relative mRNA expression of *p65* in HSC3 and SCC4 cells transfected with different sip65 siRNAs. We selected siRNA 665 to target *p65*. Cells were transfected with siRNA 665 prior to stimulation with LPS or poly (I:C). (Top) Relative mRNA expression of *HIF1A* and *VEGF* in HSC3 and SCC4 cells following sip65 665 transfection. (**B**) Relative mRNA expression of *HIF1A* and *VEGF* in HSC3 and SCC4 cells cultured in 1% O_2_ for 6 h with 50 ng/mL TNF-α or 50 μM BAY 11–7082. (**C**) Western blot analysis of changes in HIF-1α and VEGF protein levels. (**D**) Relative mRNA expression of *HIF1A* and *VEGF* in HSC3 and SCC4 cells cultured in 20% O_2_ for 6 h with 50 ng/mL TNF-α or 50 μM BAY 11–7082. (**E**) VEGF protein levels in HSC3 and SCC4 cells that were treated with 10 μg/mL poly (I:C), transfected with siTLR3 or sip65, and then treated once more with 10 μg/mL poly (I:C). (**F**) VEGF protein levels in HSC3 and SCC4 cells that were treated with 10 μg/mL LPS, transfected with siTLR4 or sip65, and then treated once more with 10 μg/mL LPS. Error bars indicate SE (*p ≤ 0.05; **p ≤ 0.01; ***p ≤ 0.001). NC, negative control; nor, normoxic conditions; hypo, hypoxic conditions.

It was previously reported that NF-κB could regulate *HIF-1* expression in endometrial carcinoma and malignant lymphoma [27, 28]. Given that our results indicated both HIF-1 and NF-κB were upregulated through the TLR3 and TLR4 pathways in OSCC cell lines (Figures 3–4), we investigated whether NF-κB could also regulate *HIF-1* expression in OSCC cells. After evaluating the knockdown efficiency of three siRNAs against p65 (data not shown), we demonstrated that a highly efficient siRNA (si-p65 665) could inhibit LPS- and poly (I:C)-induced *HIF-1α* and *VEGF* expression in both HSC3 and HSC4 cells (Figure 5A).

We next incubated OSCC cells under hypoxic conditions (1% O_2_ for 6 h in the presence or absence of either TNF-α, an NF-κB stimulator, or BAY 11–7082, an NF-κB inhibitor). The results indicated that TNF-α enhanced, but BAY 11–7082 inhibited, hypoxia-induced *HIF-1α* and *VEGF* expression at both the mRNA and protein levels (Figure 5B–5C). Similarly, when cells were cultured under normoxic conditions (20% O_2_) for 6 h, TNF-α induced expression of *HIF-1α* and *VEGF* (both mRNA and protein), whereas expression was inhibited by BAY 11–7082 (Figure 5D, Supplementary Figure 1A). These results showed that even under normoxic conditions, TNF-α could induce expression of *HIF-1α* and *VEGF*, which was inhibited by BAY 11–7082. These data strongly suggested that NF-κB was involved in regulation of TNF-mediated *HIF-1α* and *VEGF* expression in OSCC cells.

**Figure 6:**
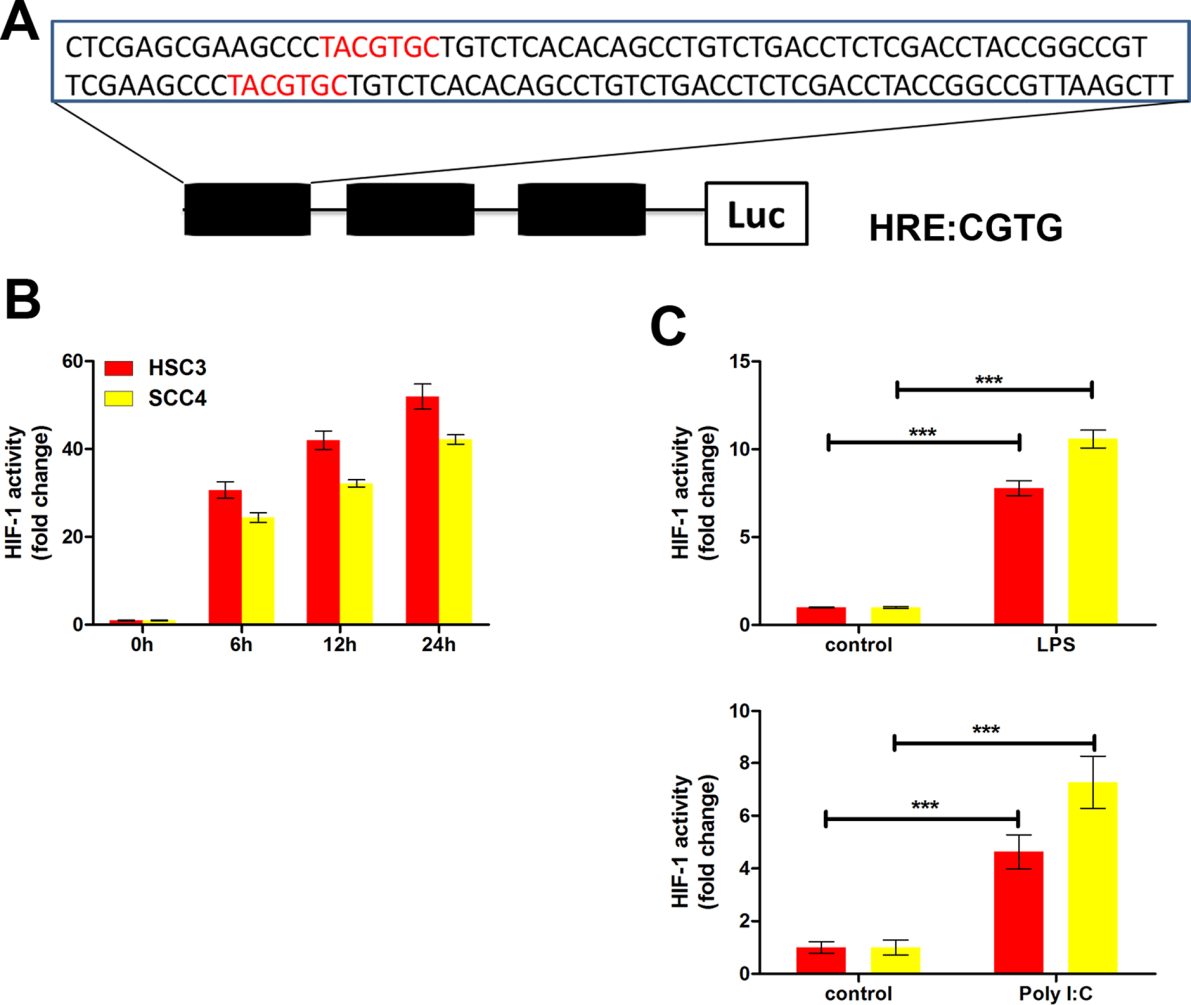
The TLR3 and TLR4 pathways regulate HIF-1 activity in HSC3 and SCC4 cells (**A**) Three HRE sequences (3xHRE), each containing two HRE binding sites, were inserted into a pGL6-luciferase reporter plasmid. (**B**) Luciferase reporter assay in HSC3 and SCC4 cells transfected with the 3xHRE reporter plasmid and cultured under hypoxic conditions for 6, 12, and 24 h. (**C**) Luciferase reporter assay of HSC3 and SCC4 cells transfected with the 3xHRE reporter plasmid and cultured under normoxic conditions with 10 μg/mL LPS or poly (I:C) for 24 h. Error bars indicate SE (*p ≤ 0.05; **p ≤ 0.01; ***p ≤ 0.001).

We also examined the impact of poly (I:C) and LPS on the expression of VEGF and HIF-1α. Consistent with the mRNA levels (Figure 2), both poly (I:C) and LPS increased VEGF expression under normoxic conditions, whereas expression was inhibited by TLR siRNAs (TLR3 and TLR4 siRNA) and p65 siRNA (Figure 5E, 5F). Similarly, expression of both HIF-1α and VEGF was induced by hypoxia, but this induction was inhibited by siRNAs against TLR3 and TLR4 (Supplementary Figure 1B–1C). Finally, to further explore the roles of TLRs in HIF-1 regulation, we inserted a sequence containing three copies of the HIF-1-binding hypoxia response element (HRE) into a pGL6 plasmid (pGL6-3xHRE) (Figure 6A). When HSC3 and SCC4 cells were transfected with pGL6-3xHRE and then cultured under hypoxic conditions, luciferase activity was greatly increased (Figure 6B). Consistent with these results, when cells transfected with pGL6-3xHRE were treated with LPS or poly (I:C) under normoxic conditions, luciferase activity was also significantly increased (Figure 6C), which provided additional evidence that TLR signaling could regulate HIF-1α activity in OSCC cells. Collectively, these data demonstrated that *TLR3* and *TLR4* were expressed in OSCC and that the TLR activators LPS and poly (I:C) could regulate *HIF-1α* and *VEGF* expression through NF-κB.

### Hypoxia induces *TLR3* and *TLR4* expression through HIF-1

**Figure 7:**
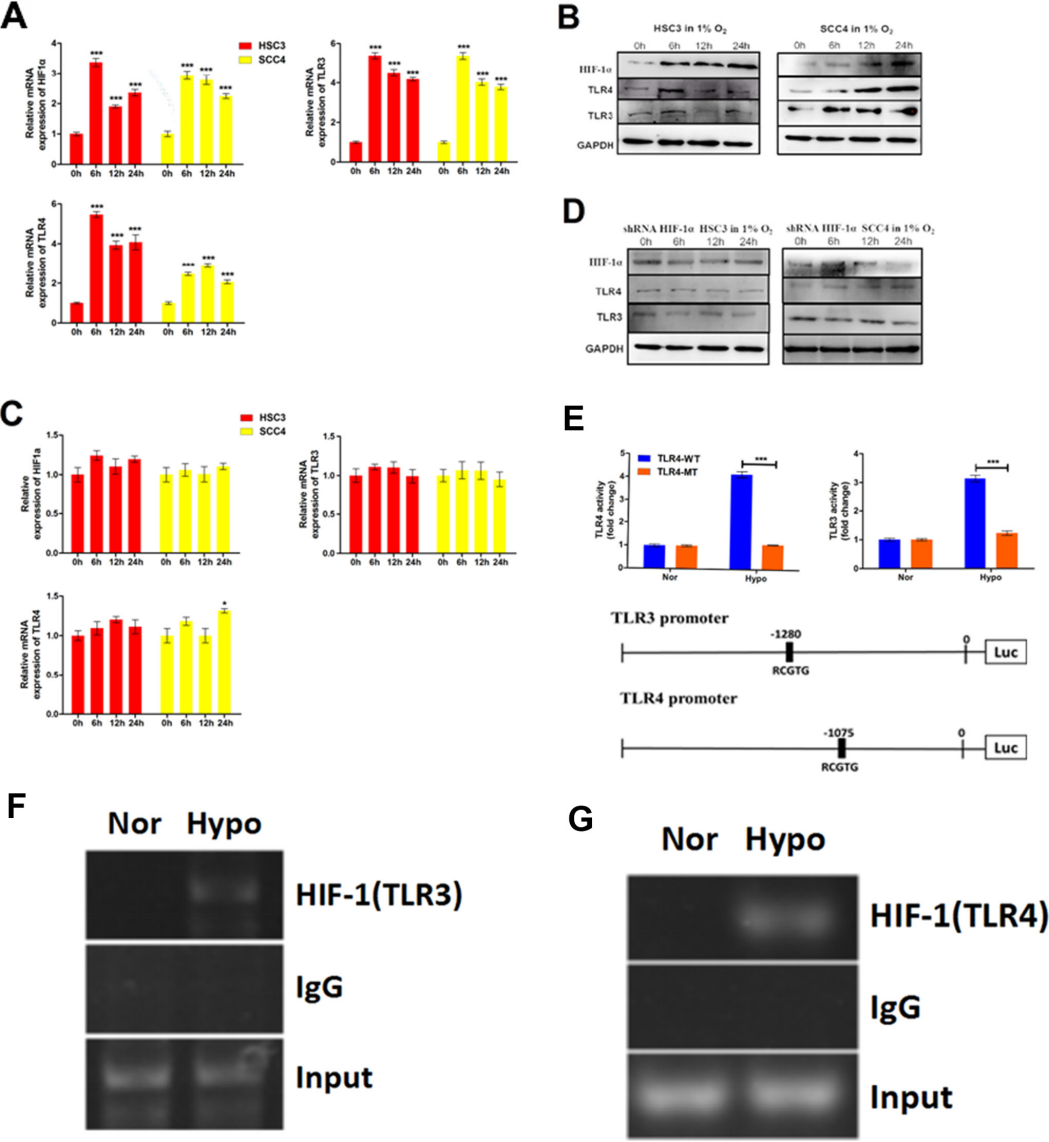
Hypoxia induces TLR3 and TLR4 expression via HIF-1 (**A**) Relative mRNA expression of *HIF1A*, *TLR3*, and *TLR4* in HSC3 and SCC4 cells cultured in 1% O_2_ for 6, 12, and 24 h. (**B**) Expression of HIF-1α, TLR3, and TLR4 in HSC3 and SCC4 cells cultured in 1% O_2_ for 6, 12, and 24 h. (**C–D**) Expression of HIF-1α, TLR3, and TLR4 mRNA (C) and protein (D) in HSC3 and SCC4 cells transfected with or without HIF-1α shRNA and cultured in 1% O_2_ for 6, 12, and 24 h. (**E**) Luciferase reporter assay in HSC3 and SCC4 cells transfected with wild-type (WT) or mutant (MT) *TLR3* and *TLR4* promoter luciferase reporters and cultured under 20% O_2_ (normoxic, nor) or 1% O_2_ (hypoxic, hypo) conditions for 24 h. (**F**) ChIP assay of the TLR3 promoter (−1280 bp). HSC3 cells were cultured in 1% O_2_ or 20% O_2_ for 24 h. (**G**) ChIP assay of the TLR4 promoter (−1075 bp). HSC3 cells were cultured in 1% O_2_ or 20% O_2_ for 24 h. Error bars indicate SE (*p ≤ 0.05; **p ≤ 0.01; ***p ≤ 0.001).

To determine whether hypoxia regulated *TLR3* and *TLR4* expression, we exposed two OSCC cell lines to hypoxic conditions for varying times. Both *TLR3/TLR4* mRNA and protein levels were upregulated under hypoxic conditions (Figure 7A–7B). To evaluate the possible role of HIF-1 in the hypoxia-induced upregulation of *TLR3* and *TLR4*, we established HSC3 and SCC4 stable cell lines with low HIF-1α expression using a HIF-1α short hairpin RNA (shRNA) lentiviral vector (data not shown). Hypoxia-induced upregulation of *HIF-1* mRNA and protein was inhibited in these cells (Figure 7C–7D), and the upregulation of *TLR3/TLR4* mRNA and protein by hypoxia suppressed, indicating that HIF-1 was a key mediator of the hypoxia-induced upregulation of *TLR3* and *TLR4* mRNA and protein.

To investigate whether HIF-1 could directly regulate *TLR3* and *TLR4* expression, we analyzed the promoter sequences of each gene and identified possible functional HIF-1-binding HRE sequences (5′-RCGTG-3′) located upstream of the *TLR3* and *TLR4* promoters, respectively. This raised the possibility that these HREs were directly involved in HIF-1-mediated regulation of *TLR3* and *TLR4*. To test this hypothesis, we generated luciferase reporters driven by either wild-type or HRE-mutated (e.g., from 5′-RCGTG-3′ to 5′-RAAAG-3′) *TLR3* and *TLR4* promoters, respectively (Figure 7E). Consistent with this hypothesis, hypoxia induced luciferase reporter activity in the presence of the wild-type *TLR3* or *TLR4* promoters, whereas HRE mutations in the *TLR3* and *TLR4* promoters impaired or abolished luciferase activity under hypoxic conditions (Figure 7E). These results strongly suggested that HIF-1 directly regulated *TLR3* and *TLR4* promoter activity. Finally, we used a chromatin immunoprecipitation (ChIP) assay to examine the actual binding sites of HIF-1 within the promoter regions of *TLR3* and *TLR4*. Our results revealed that sequences at −1280 bp of the *TLR3* promoter and at −1075 bp of the *TLR4* promoter were functional HIF-1-binding sites (Figure 7F–7G), providing further support for a direct role of HIF-1 in the regulation of *TLR* expression.

### Hypoxia induces the expression of NF-κB and pro-inflammatory cytokines via HIF-1

**Figure 8:**
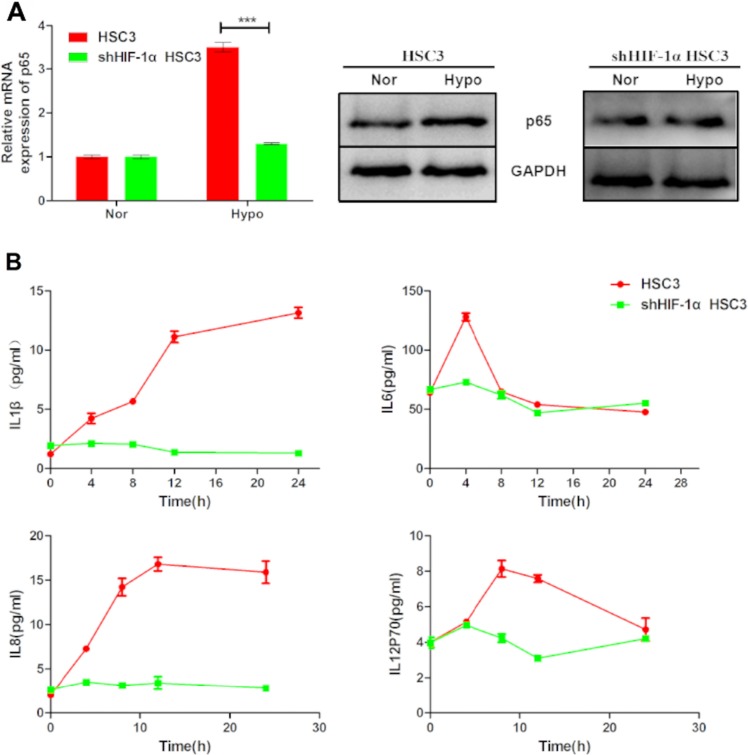
Hypoxia upregulates *p65* and pro-inflammatory cytokine expression via HIF-1α (**A**) Expression of *p65* mRNA and p65 protein in HSC3 cells transfected with or without HIF-1α shRNA under normoxic (nor) or hypoxic (hypo) conditions for 24 h. (**B**) IL-1β, IL-6, IL-8, and IL-12p70 released by non-transfected and HIF-1α shRNA-transfected HSC3 cells cultured under normoxic (nor) or hypoxic (hypo) conditions for 4, 8, 12, and 24 h. Error bars indicate SE (*p ≤ 0.05; **p ≤ 0.01; ***p ≤ 0.001).

NF-κB is a transcription factor that regulates the expression of inflammatory cytokines. It can be activated by many factors including cytokines, ultraviolet irradiation, and bacterial or viral infections. Interestingly, hypoxia induced *p65* expression in HSC3 and SCC4 cells (Figure 8A). Moreover, our results showed that this induction was diminuished by HIF-1α shRNA (Figure 8A), suggesting that hypoxia-induced *p65* expression was dependent on HIF-1 in OSCC cells. Finally, consistent with the role of NF-κB in inflammation, increased expression of the inflammatory cytokines IL-1β, IL-6, IL-8, and IL-12p70 was also observed in cells cultured under hypoxic conditions (Figure 8B).

### *In vivo* analysis of human OSCC using a transplantation model in nude mice

**Figure 9:**
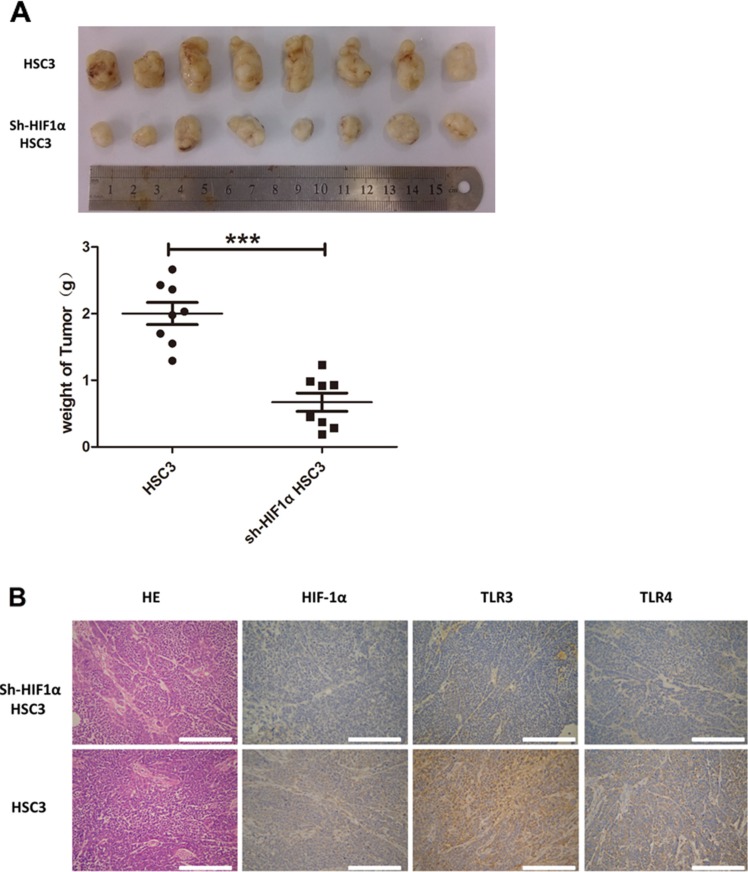
*In vivo* study of human OSCC using a transplantation model in nude mice (**A**) HSC3 and sh-HIF1α HSC3 were injected into nude mice. After 30 days, tumors were collected and weighed. (**B**) IHC analyses of HIF1α, TLR3, and TLR4 expression in tumor tissue (200×) (*p ≤ 0.05; **p ≤ 0.01; ***p ≤ 0.001).

To explore the relationship between HIF-1 and TLRs, we established an OSCC transplantation model in nude mice using normal and HIF-1α shRNA-treated HSC3 cells. Inhibition of *HIF-1α* expression by shRNA significantly reduced tumor growth in mice (Figure 9A). Importantly, HIF-1α expression in tumors was positively correlated with TLR3 and TLR4 expression (Figure 9B). These data confirmed the results of the *in vitro* studies and suggested that TLR and HIF-1 mutually regulated each other in OSCC cells.

## DISCUSSION

**Figure 10:**
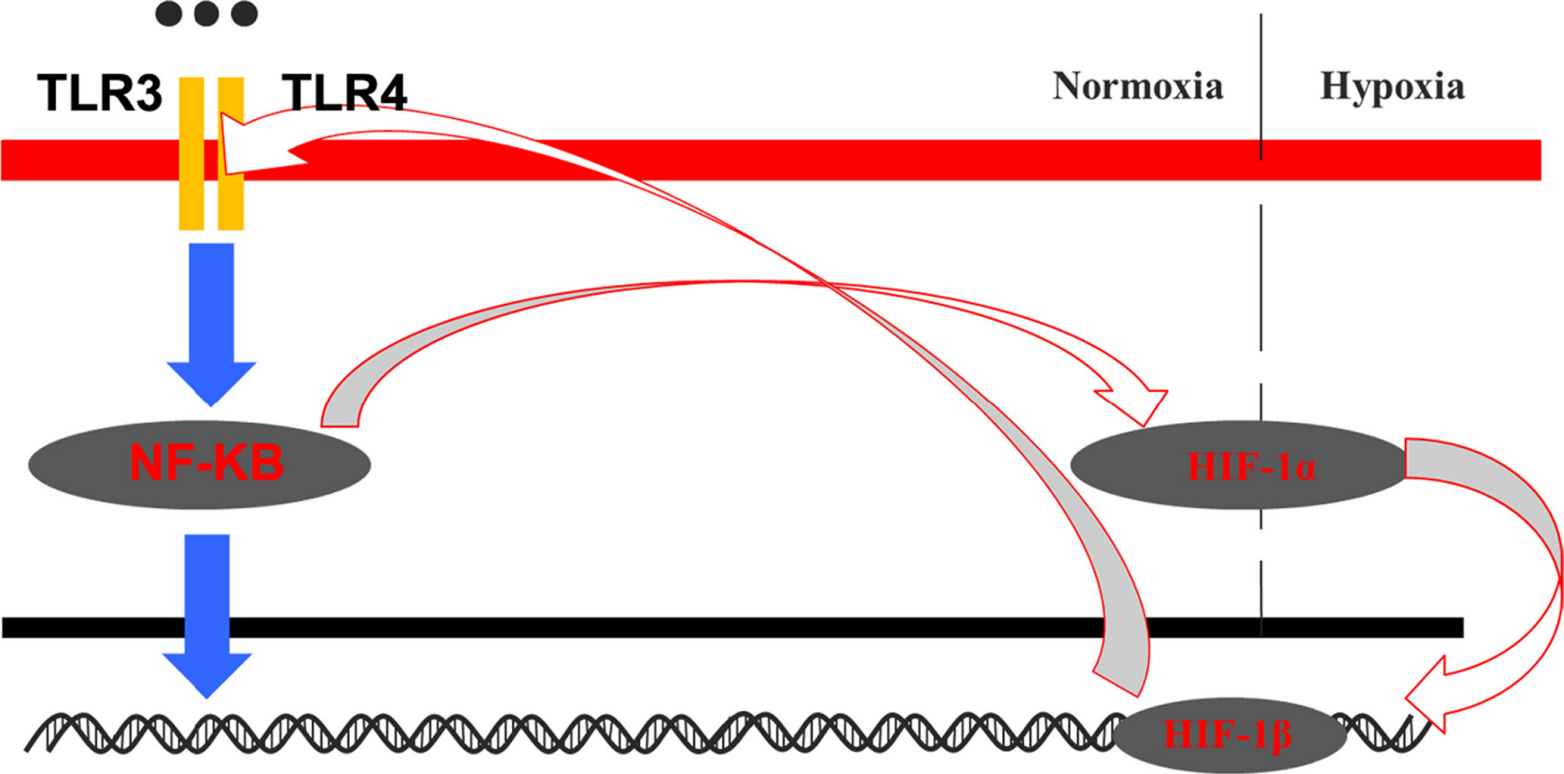
Model for the crosstalk between the HIF-1 and TLR-NF-κB pathways in OSCC

We demonstrated that upregulation of *HIF-1α* was directly correlated with activation of *TLR3* and *TLR4* through NF-κB signaling. Importantly, our results suggested that HIF-1 could directly regulate TLR/NF-κB signaling in OSCC cells, suggesting the existence of a positive feedback loop between HIF-1 and TLR/NF-κB signaling in OSCC cells (Figure 10). Cross-talk between HIF-1 and TLR/NF-κB signaling may contribute to OSCC initiation and progression.

TLRs are crucial components of the innate immune response to bacterial and viral pathogens [29]. Recent studies have revealed that they can be “double-edged swords” because they might also be expressed in tumor cells [30]. TLR3 was previously detected in papillary thyroid, lung, and breast cancer [31]. TLR4 has been detected in both gastric and colorectal cancer [32]. We demonstrated that *TLR3* and *TLR4* are highly expressed in OSCC (Figure 1A) which is consistent with previous studies [24, 25, 33]. The expression of *TLR4* has been correlated with resistance of human OSCC to chemotherapy [34], and *TLR2* expression was shown to be highly correlated with tumor progression [35]. Here, we screened for expression of *TLR2, −3, −4, −7*, and *-9*. *TLR2* was detected but at a much lower level than *TLR3* and *TRL4* (data not shown). Thus, TLR3 and TLR4 might be more important for OSCC progression than the other TLRs. HIF-1α may be directly regulated by TLR/NF-κB signaling since the *HIF1A* promoter contains an active NF-κB binding site in position 197/188, upstream of the transcription start site [36]. Although it has been shown that the TLR-myeloid differentiation primary response 88 (MyD88)-NF-κB signaling pathway could activate NF-κB in immune cells, there have been few reports regarding the functions of TLRs in OSCC. Similarly, although it was reported that HIF-1 could up-regulate *TLR4* expression in pancreatic cancer cells under hypoxic conditions [39], to our knowledge this is the first report that HIF-1 can upregulate *TLR3* and *TLR4* in OSCC cell lines under hypoxia.

Given that HIF-1 regulates more than 100 downstream genes such as *VEGF, Bcl-2*, and *survivin*, activation of HIF-1 in oral tumor cells by hypoxia and inflammatory signals will induce target gene expression, thereby promoting cell survival and metastasis. Moreover, our data suggest that bacteria, viruses, fungi, and protozoa in the microenvironment could activate the HIF-1 pathway. Hypoxia could result in increased release of IL-1β, IL-6, IL-8, and IL-12P70 in HSC3 and SCC4 cells and upregulation of the mRNA levels of these cytokines through HIF-1 (Figure 8). In our study, the baseline levels of IL-1β, IL-8, and IL-12P70 in HSC3 and SCC4 cells were much lower than those of IL-6. The release of inflammatory cytokines from OSCC cells could promote cell transformation, survival, proliferation, and metastasis. These cytokines are upregulated by NF-κB, which is further upregulated under hypoxic conditions by HIF-1.

Hypoxia and inflammation are two typical features of the microenvironment in solid tumors [40]. Our studies not only demonstrated that *TLR3* and *TLR4* are highly expressed in OSCC, but also that TLR3/TLR4–NF-κB signaling is active in oral tumor cells. This signaling pathway forms a TLR3/TLR4–NF-κB–HIF-1 loop that is closely associated with hypoxia and inflammation in the tumor and leads to tumor cell survival, proliferation, angiogenesis, and metastasis. Our findings will help with the development of novel and more effective therapeutic strategies for OSCC based on HIF-1 and TLR/NF-κB inhibition.

## MATERIALS AND METHODS

### Ethics statement

All experiments involving human samples were performed in accordance with the ethical standards and according to the Declaration of Helsinki. All animal work was conducted according to national and international guidelines. Institutional review board approval was gained for this study from Nanjing Stomatological Hospital Ethics Committee, approval number: 2015NL-009(KS).

### Cell culture and reagents

Human OSCC cell lines (HSC3 and SCC4) were obtained from the Cell Bank of the Chinese Academy of Sciences (Shanghai, China). Cells were grown in monolayer cultures in Dulbecco's Modified Eagle's Medium (Gibco, Grand Island, NY, USA) supplemented with 10% fetal bovine serum (Sigma-Aldrich, St. Louis, MO, USA), 100 μg/mL streptomycin, and 100 U/mL penicillin in a humidified incubator (5% CO_2_) at 37°C. Confluent cells were trypsinized with 0.05% trypsin/0.02% EDTA.

### Hypoxic treatment

Cells were placed in a humidified hypoxia workstation (Memmert, Germany) equilibrated to 1% O_2_ and 5% CO_2_ at 37°C. The experimental chemical compounds were added to the cells immediately before placement in the hypoxia chamber. Cell lysates were collected immediately after cells had been removed from the hypoxia chamber.

### Reagents and antibodies

LPS derived from *Escherichia coli* strain 055: B5, and the NF-κB inhibitor BAY 11–7028 were purchased from Sigma-Aldrich. Recombinant human TNF-α was obtained from PeproTech. Poly (I:C) was obtained from InvivoGen. The following antibodies were used for western blotting: an anti-TLR4 rabbit polyclonal antibody (Abcam); anti-TLR3 rabbit polyclonal antibody (Cell Signaling Technology); anti-glyceraldehyde-3-phosphate dehydrogenase (GAPDH) rabbit polyclonal antibody (Bioworld Technology); anti-HIF-1α rabbit polyclonal antibody (Cell Signaling Technology), anti-VEGF rabbit polyclonal antibody (Abcam). Protein standards were obtained from SunShineBio. Horseradish peroxidase (HRP)-conjugated goat anti-rabbit immunoglobulin G (IgG) was purchased from Immunology Consultants Laboratory (Newberg, OR, USA). An anti-p65 rabbit polyclonal antibody (Cell Signaling Technology) and goat anti-rabbit IgG (Bioworld Technology) were used for immunofluorescence.

### RNA extraction and qRT-PCR

Total RNA from OSCC cells was isolated using TRIzol reagent (Invitrogen) according to the manufacturer's protocol. After RNA quantification, 2 μg of total RNA was reverse-transcribed using the cDNA Reverse Transcription kit (Takara) according to the manufacturer's instructions. QRT-PCR was performed using a StepOne Real-Time PCR System (Applied Biosystems). The mRNA expression levels of the target genes were assessed by qRT-PCR using iTaq SYBR Green Supermix with ROX (Bio-Rad) and the following cycling conditions: 95°C for 2 min, and 40 cycles of 95°C for 15s and 60°C for 45 s. The comparative threshold cycle (Ct) method was used to evaluate gene expression. The primers used in the experiments are listed in Table 1.

**Table 1:** Primers of Q-PCR

*GENE*	Forward	Reverse
*TLR2*	ATCCTCCAATCAGGCTTCTCT	GGACAGGTCAAGGCTTTTTACA
*TLR3*	TTGCCTTGTATCTACTTTTGGGG	TCAACACTGTTATGTTTGTGGGT
*TLR4*	AGACCTGTCCCTGAACCCTAT	CGATGGACTTCTAAACCAGCCA
*TLR7*	CACATACCAGACATCTCCCCA	CCCAGTGGAATAGGTACACAGTT
*TLR9*	CTGCCACATGACCATCGAG	GGACAGGGATATGAGGGATTTGG
*HIF-1α*	TTTGCTGAAGACACAGAAGCAAAGA	TTGAGGACTTGCGCTTTCAGG
*VEGF*	AGGGCAGAATCATCACGAAGT	AGGGTCTCGATTGGATGGCA
*β-actin*	CTGGGACGACATGGAGAAAA	AAGGAAGGCTGGAAGAGTGC
*p65*	ATGTGGAGATCATTGAGCAGC	CCTGGTCCTGTGTAGCCATT

### Western blotting

Cells cultured under hypoxic or normoxic conditions were harvested and lysed for 20 min using modified RIPA buffer (5 mM EDTA, 2 mM Na_3_VO_4_, 5 mM NaF, 1 mM phenylmethylsulfonyl fluoride) supplemented with protease inhibitor cocktail (Sigma-Aldrich). The supernatant (total cellular protein extract) was harvested after centrifugation for analysis. Protein concentrations were determined using a BCA assay (Micro BCA; Pierce, Rockford, IL, USA). Samples containing approximately 40 μg protein were subjected to 10% sodium dodecyl sulfate (SDS)–polyacrylamide gel electrophoresis in Tris-Tricine-SDS buffer, and then electrophoretically transferred to polyvinylidene difluoride membranes (Bio-Rad), which were then blocked for 2 h at room temperature in 5% non-fat milk. The membranes were incubated for 1 h with gentle agitation at 4°C with Abs in 2.5% non-fat milk, washed three times with Tris-buffered saline-Tween 20, and incubated with the appropriate HRP-conjugated secondary antibody diluted in secondary antibody dilution buffer (EnoGene) for 1 h at room temperature.

### Immunofluorescence microscopy analysis

For immunofluorescence staining, cells were seeded onto 12-mm diameter round glass coverslips in 24-well culture dishes. The cells were fixed with 4% paraformaldehyde for 20 min and then washed and permeabilized with 0.5% Triton X-100 in phosphate-buffered saline (PBS) for 20 min. The cells were stained with a primary anti-p65 antibody at 4°C overnight (1:1000 in 1% bovine serum albumin in PBS). Finally, the cells were incubated with the secondary antibody (fluorescein isothiocyanate-conjugated anti-rabbit) for 2 h at room temperature and the nuclei stained with diaminophenylindole (DAPI, 1:100 in PBS) for 15 min. Images were captured using an Olympus FV10i microscope (Olympus).

### Knock down of TLR3, TLR4, P65, and HIF-1α with siRNA

The siRNAs used to knock down TLR3, TLR4, and P65 expression in OSCC cells were obtained from GenePharma. We designed three sequences for each target. Cells transfected with different concentrations of each siRNA were then cultured for 24 h. The relative mRNA expression of target gene was measured by qRT-PCR. The siRNAs that achieved the most effective knock-down of the targets were selected for use in experiments. The sequences of each siRNA used are listed in Table 2. The siRNAs and non-silencing controls were transfected into cells using Lipofectamine 2000 (Invitrogen). Transfected cells in fresh media were then incubated under the appropriate experimental conditions and harvested for sample preparation.

**Table 2:** Sequences of siRNA

GENE	Target site	Sense (5′–3′)	Anti-sense (5′–3′)
*TLR4*	602	CCACCUCUCUACCUUAAUATT	UAUUAAGGUAGAGAGGUGGTT
	1332	CCCACAUUGAAACUCAAAUTT	AUUUGAGUUUCAAUGUGGGTT
	1553	GGGCUUAGAACAACUAGAATT	UUCUAGUUGUUCUAAGCCCTT
*TLR3*	1757	GUCCCAUUUAUUUCCUAAATT	UUUAGGAAAUAAAUGGGACTT
	2025	GCGCUUUAAUCCCUUUGAUTT	AUCAAAGGGAUUAAAGCGCTT
	2658	GGAGAUUCCAGAUUAUAAATT	UUUAUAAUCUGGAAUCUCCTT
*P65*	755	GGGAUGAGAUCUUCCUACUTT	AGUAGGAAGAUCUCAUCCCTT
	1455	GCUGCAGUUUGAUGAUGAATT	UUCAUCAUCAAACUGCAGCTT
	1707	CCUCCUUUCAGGAGAUGAATT	UUCAUCUCCUGAAAGGAGGTT
Negative control		UUCUCCGAACGUGUCACGUdTdT	ACGUGACACGUUCGGAGAAdTdT

### Plasmid construction and luciferase assays

The firefly luciferase reporter plasmid was purchased from Promega (Madison, WI, USA). We designed forward and reverse oligonucleotides containing three copies of the HRE sequence (5′-GTACGTACT-3′) on each end using the NheI and HindIII restriction sites (New England Biolabs, Beverly, MA, USA). The oligonucleotides were annealed and ligated using the NheI and HindIII sites to create Phre-Luc.

Wild type or mutant *TLR3* and *TLR4* promoters were inserted into each plasmid. Following ligation, transformation, and screening, all plasmids were verified by sequencing.

Cells were transfected with plasmids using Lipofectamine 2000 (Invitrogen) according to the manufacturer's protocol. Luciferase expression was measured using a One-Glo Luciferase Assay kit (Promega) and the luminescence function of a GloMax Multi microplate reader (Promega). The data are presented as the mean and standard deviation (SD) of three experimental replicates.

### Enzyme-linked immunosorbent assays

Cytokine levels were quantified using human VEGF, IL-1βa, IL-6, and IL-8 enzyme-linked immunosorbent assays (ELISA) kits (R&D Systems, Minneapolis, MN, USA) according to the manufacturer's instructions. All standards and samples were tested in triplicate.

### ShRNA transfection and viral transduction

Recombinant lentivirus pLvx-Luciferase-puro-HIF1a-1+2+3+4 was generated by transfecting HEK293T cells. After 48 h, media containing the viral particles was harvested and passed through a 0.45-μm filter (Millipore, New Bedford, MA, USA). For transduction, 10 μL of the supernatant containing lentivirus was added to HSC-3 cells, which were maintained in 2 mL complete culture media. In addition, 8 μg/mL of polybrene (Sigma-Aldrich) was also added to aid the transduction. After 24 h, the media were replaced by fresh media supplemented with 10% FBS containing 0.5 μg/mL puromycin (Sigma-Aldrich). Cells were maintained in puromycin-containing medium for selection of stable transfectants. The shRNA sequences are listed in Table 3.

**Table 3:** shRNA sequences of HIF-1α

shRNA1	GGGATTAACTCAGTTTGAACT
shRNA2	GCCGAGGAAGAACTATGAACA
shRNA3	GCATTGTATGTGTGAATTACG
shRNA4	GCTGGAGACACAATCATATCT

### Human OSCC transplantation model in nude mice

All mice were maintained under specific pathogen free conditions. The experimental protocols were approved by the Ethics Committee of Nanjing Stomatological Hospital. Immunoincompetent nude mice were injected subcutaneously in the flank with either 100 μL HSC3 or sh-HIF1α HSC3 (1 × 10^7^/mL; 8 mice/group). After 30 days, tumors were collected and weighed to compare tumor growth.

### Immunohistochemistry

Xenograft tumors were fixed with 10% formalin, embedded in paraffin, sectioned (5-μm thickness), dewaxed with xylene, and then hydrated in a graded series of ethanol. DAKO was used with the HIF-1α antibody (1:200, Abcam), TLR3 antibody (1:100, Abcam), and TLR4 antibody (1:300, Abcam). Sections were counterstained with Mayer's hematoxylin, dehydrated through graded ethanol into xylene, and then mounted.

### Chromatin immunoprecipitation assays

HSC3 cells were cultured in either 20% or 1% O_2_ for 24 h. The cells were then cross-liked in 1% formaldehyde for 10 min, quenched in 0.125 M glycine, and lysed with SDS lysis buffer. Chromatin was sheared by sonication (Diagenode). We used a ChIP kit (One Step, Abcam) and a ChIP grade primary antibody against HIF-1α (Abcam). We used the following primers for the *TLR3* promoter: Forward 5′-CAAGCCTGGGTAATCGTGTTTG-3′; Reverse 5′-GCAACCCTCTGAGGTAGACTG-3′. The primers for the *TLR4* promoter were the following: Forward 5′-CTCACTTCTGAGTACGTATCC-3′; Reverse 5′-GTATAGATCAAAGCCTCCTTCC-3′.

### Statistical analysis

All data are presented as the mean ± SD or standard error (SE) of the mean, and *n* corresponds to the number of experiments. Statistical differences between groups were evaluated using paired Student's *t*-tests or one-way analysis of variance followed by a Student's *t*-test with Bonferroni correction as indicated. Statistical analyses were performed using the GraphPad Prism 5.0 software (version 5.01 for Windows, GraphPad Software, Inc., San Diego CA, USA). A *p* < 0.05 was considered statistically significant.

## SUPPLEMENTARY MATERIALS FIGURE


