# Supplementary material for: Crosstalk between the HIF-1 and Toll-like receptor/nuclear factor-κB pathways in the oral squamous cell carcinoma microenvironment
Source: Oncotarget. 2016 May 12;7(25):37773–89. doi: 10.18632/oncotarget.9329 (PMC5122348; doi:10.18632/oncotarget.9329)
Supplement: Supplementary file 1 [file oncotarget-07-37773-s001.pdf]

Crosstalk between the HIF-1 and Toll-like receptor/nuclear factor-κB pathways in the oral squamous cell carcinoma microenvironment

Supplementary Materials

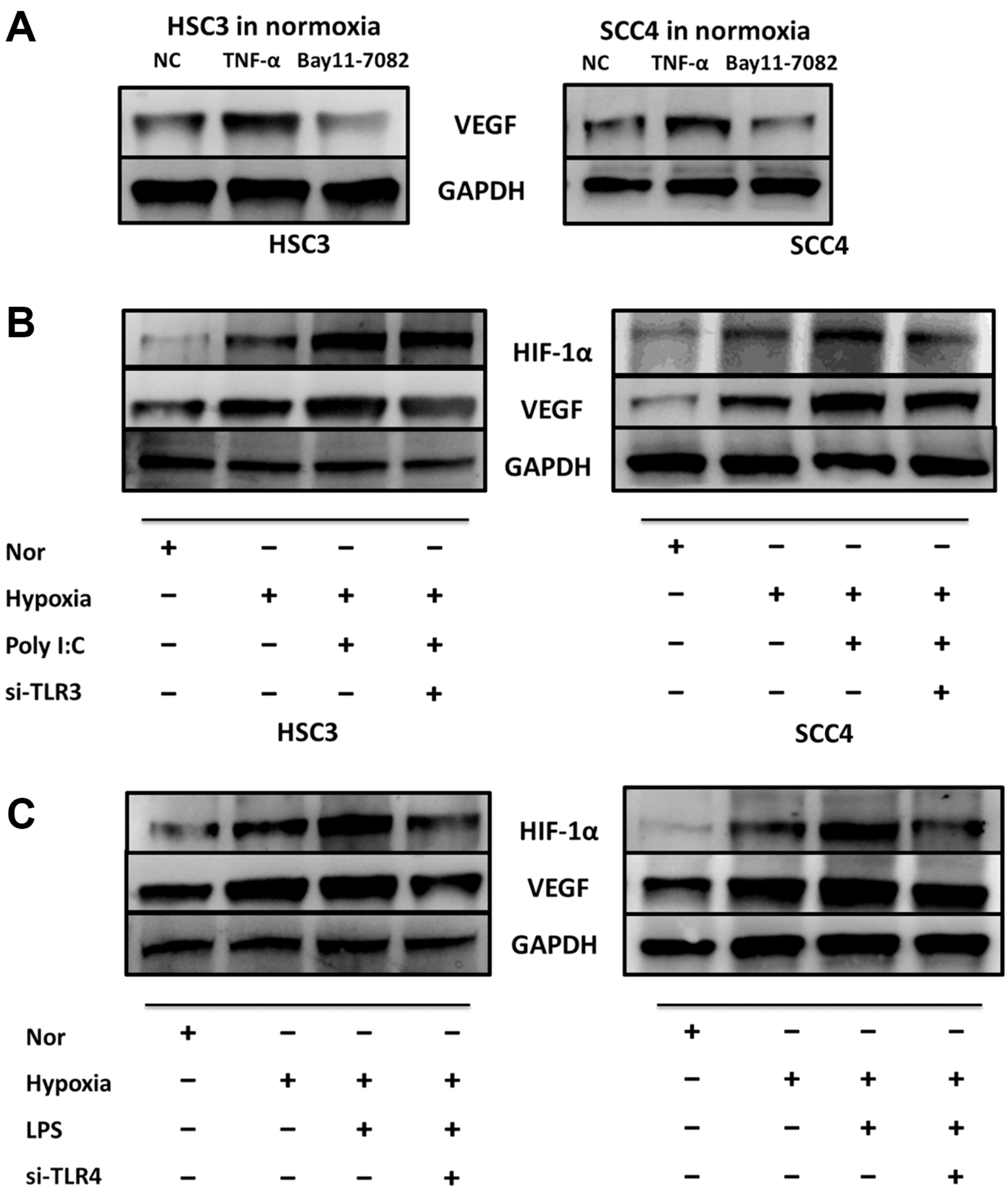

Supplementary Figure S1: TLR-NF-κB pathway regulation of HIF-1 expression in HSC3 and SCC4 cells. (**A**) VEGF expression in HSC3 and SCC4 cells cultured in 20% O_2 for 6 h in the presence of 50 ng/mL TNF-α or 50 µM BAY 11-7082. (**B**) HIF1A and VEGF expression in cells cultured under hypoxic conditions (1% O_2) for 12 h. OSCC cells were transfected with 40 nM siTLR3 2658 prior to treatment with 10 µg/mL poly(I:C). (**C**) HIF1A and VEGF expression in cells cultured under hypoxic conditions (1% O_2) for 12 h. OSCC cells were transfected with 20 nM siTLR4 1332 prior to treatment with 10 µg/mL LPS.
